# Cognitive and motor dual task gait training improve dual task gait performance after stroke - A randomized controlled pilot trial

**DOI:** 10.1038/s41598-017-04165-y

**Published:** 2017-06-22

**Authors:** Yan-Ci Liu, Yea-Ru Yang, Yun-An Tsai, Ray-Yau Wang

**Affiliations:** 1 Department of Physical Therapy and Assistive Technology, National Yang-Ming University, Taipei, ROC Taiwan; 2 Division of Neural Repair, Department of Neurosurgery, Neurological Institute, Taipei Veterans General Hospital, Taipei, Taiwan

## Abstract

This study investigated effects of cognitive and motor dual task gait training on dual task gait performance in stroke. Participants (n = 28) were randomly assigned to cognitive dual task gait training (CDTT), motor dual task gait training (MDTT), or conventional physical therapy (CPT) group. Participants in CDTT or MDTT group practiced the cognitive or motor tasks respectively during walking. Participants in CPT group received strengthening, balance, and gait training. The intervention was 30 min/session, 3 sessions/week for 4 weeks. Three test conditions to evaluate the training effects were single walking, walking while performing cognitive task (serial subtraction), and walking while performing motor task (tray-carrying). Parameters included gait speed, dual task cost of gait speed (DTC-speed), cadence, stride time, and stride length. After CDTT, cognitive-motor dual task gait performance (stride length and DTC-speed) was improved (p = 0.021; p = 0.015). After MDTT, motor dual task gait performance (gait speed, stride length, and DTC-speed) was improved (p = 0.008; p = 0.008; p = 0.008 respectively). It seems that CDTT improved cognitive dual task gait performance and MDTT improved motor dual task gait performance although such improvements did not reach significant group difference. Therefore, different types of dual task gait training can be adopted to enhance different dual task gait performance in stroke.

## Introduction

Cognitive-motor and motor dual tasks play important roles in daily life: walking while talking, using a mobile phone, carrying a bag or watching traffic. Previous studies have indicated, however, that performing two tasks simultaneously may negatively impact gait performance^1–3^. Dual task interference impacting gait performance has been observed not only in healthy subjects^4–6^, but also in subjects with neurological disorders^4, 7–10^. In stroke individuals, reductions in speed, cadence, and stride length, as well as increases in stride time during cognitive-motor dual tasking have been reported^4, 8–10^. In addition, Yang *et al*. found that stroke subjects had more difficulty executing motor dual tasks compared to healthy adults^11^. Diminished capacity for dual task performance and reduced ability to adapt to changing environments may limit stroke individuals’ ability to return to the community. Accordingly, improving walking ability in dual task situations is an important goal, especially for chronic stroke subjects with limited community ambulation.

A crucial principle in motor learning is the training of specific concepts using frequent repetitions of task-specific exercises to improve task performance^12^. It has been suggested that dual task training might have greater efficacy for improving dual task performance compared to single task training^13–15^. Subramaniam *et al*.^16^ and Lee *et al*.^17^ found significant improvements in balance ability and cognitive-motor interference under dual task conditions after combined balance and cognitive training. Regarding the dual task gait training for stroke patients, most studies emphasized its effects on single task performance (e.g. walking)^18–20^. An *et al*. demonstrated significant improvements in 10-meter walking test and 6-minute walking test after 8 weeks of treadmill with cognitive task training^18^. Kim *et al*. reported the improvement of single cognitive task (the Stroop test) and walking abilities (timed up and go test and 10-mter walking test) after 4 weeks of cognitive dual task gait training^19^. Only two studies have investigated the effects of dual task gait training on dual task gait performance in individuals with stroke. Yang *et al*., found the gait speed, cadence, stride time, and stride length were improved during motor dual task walking (tray-carrying task while walking) after 4 weeks of motor dual task gait training^21^. Plummer *et al*. also reported improvement in cognitive dual task gait speed after 12 sessions of gait training with additional cognitive task (cognitive dual task gait training)^22^. Whether different (cognitive and motor) dual task gait training results in different effects on dual task gait performance is not immediately known. However, such information can establish effective training protocol for different dual task gait performance for stroke patients. Therefore, the present study was undertaken to evaluate the effects of cognitive and motor dual task gait training on different dual task gait performance tests in individuals with stroke.

## Results

**Figure 1:**
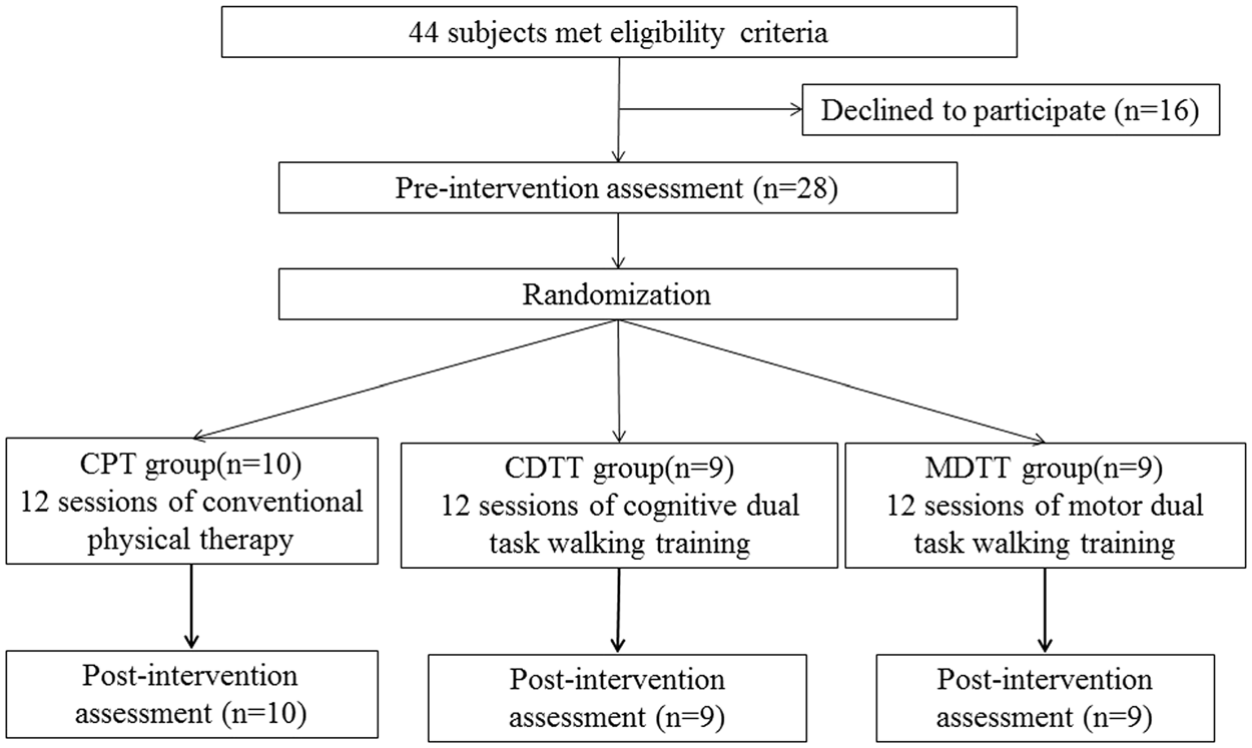
Study flow chart. Abbreviations: CPT, conventional physical therapy; CDTT, cognitive dual task gait training; MDTT, motor dual task gait training.

Twenty-eight participants were randomly assigned to the conventional physical therapy (CPT) group (n = 10), cognitive dual task gait training (CDTT) group (n = 9), or motor dual task gait training (MDTT) group (n = 9). Mean age was 50.2 ± 11.2 (range, 28–66) years old and mean period after onset was 41.1 ± 40 (range, 8–222) months. None of the participants reported any adverse events or withdrew from the study (Fig. 1). No significant differences between groups were found for baseline demographic characteristics (Table 1). Similarly, no significant differences between groups were found for any of the outcome measures at the pre-intervention assessment.

**Table 1:** Demographic characteristics of participants.

	CPT group (n = 10)	CDTT group (n = 9)	MDTT group (n = 9)	p value
Age (years)^a^	50.8 ± 13.5	51.0 ± 7.1	48.8 ± 11.7	0.91
Post stroke period (months)^a^	49.8 ± 59.8	36.4 ± 14.6	36.2 ± 25.7	0.72
Gender (male/female)^b^	8/2	8/1	8/1	0.81
Stroke type (I/H)^b^	7/3	4/5	5/4	0.53
Hemiparetic side (left/right)^b^	4/6	5/4	4/5	0.32
MMSE^a^	27.6 ± 2.5	27.7 ± 2.26	27.2 ± 1.9	0.58

Abbreviations: I, ischemic; H, hemorrhage; MMSE, mini-mental state examination; CPT, conventional physical therapy; CDTT, cognitive dual task gait training; MDTT, motor dual task gait training.

^a^Values are mean ± SD, ^b^Values are frequency.

p value, intergroup difference.

**Figure 2:**
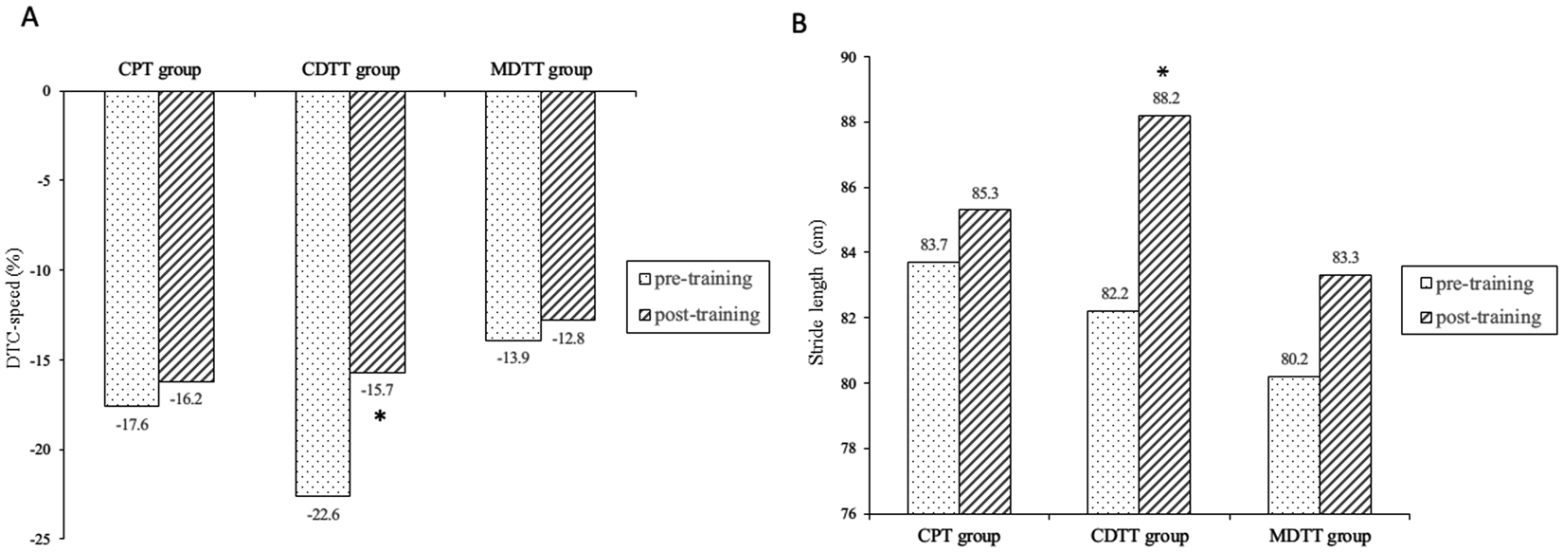
Cognitive dual task gait performance after different training protocols. (**A**) Dual task cost-speed, (**B**). Stride length*, significant intra-group difference p < 0.05. Abbreviations: CPT, conventional physical therapy; CDTT, cognitive dual task gait training; MDTT, motor dual task gait training. DTC-speed, dual task cost of speed.

Table 2 shows the measured gait outcomes while performing serial subtraction (cognitive-motor dual task) at pre- and post-intervention for three training groups. In the CDTT group, stride length increased significantly (p = 0.021) and the dual task cost of gait speed (DTC-speed) improved by 6.9% (p = 0.015) compared to pre-training (Fig. 2).

**Table 2:** Cognitive dual task gait performance after different training protocols.

	CPT group (n = 10)		CDTT group (n = 9)		MDTT group (n = 9)	
	Pre	Post	Pre	Post	Pre	Post
Speed (cm/sec)	62.1 ± 19.9	60.1 ± 20.7	56.4 ± 18.0	63.4 ± 20.6	62.4 ± 13.8	63.8 ± 13.7
Change values^a^		−2.0 ± 9.4		6.9 ± 11.1		1.4 ± 4.0
Dual task cost-speed (%)	−17.6 ± 12.2	−16.2 ± 9.3	−22.6 ± 11.0	−15.7 ± 11.3*	−13.9 ± 12.4	−12.8 ± 14.1
Change values^a^		1.4 ± 11.4		6.9 ± 6.4		1.1 ± 5.5
Cadence (step/min)	83.6 ± 14.6	83.7 ± 14.6	81.2 ± 13.5	86.1 ± 12.9	93.7 ± 11.7	93.9 ± 10.8
Change values^a^		0.1 ± 3.9		4.8 ± 10.5		0.2 ± 3.2
Stride time (sec)	1.47 ± 0.25	1.42 ± 0.19	1.52 ± 0.27	1.43 ± 0.22	1.30 ± 0.16	1.29 ± 0.14
Change values^a^		−0.05 ± 0.15		−0.09 ± 0.20		−0.01 ± 0.06
Stride length (cm)	83.7 ± 18.7	85.3 ± 17.6	82.2 ± 20.2	88.2 ± 21.4*	80.2 ± 17.2	83.3 ± 17.5
Change values^a^		1.6 ± 6.3		5.9 ± 5.9		3.1 ± 3.7

Abbreviations: CPT, conventional physical therapy; CDTT, cognitive dual task gait training; MDTT, motor dual task gait training.

Values are mean ± SD.

*p < 0.05 for intra-group difference.

^a^Change values were calculated by subtracting the pre-training data from the post-training data.

**Figure 3:**
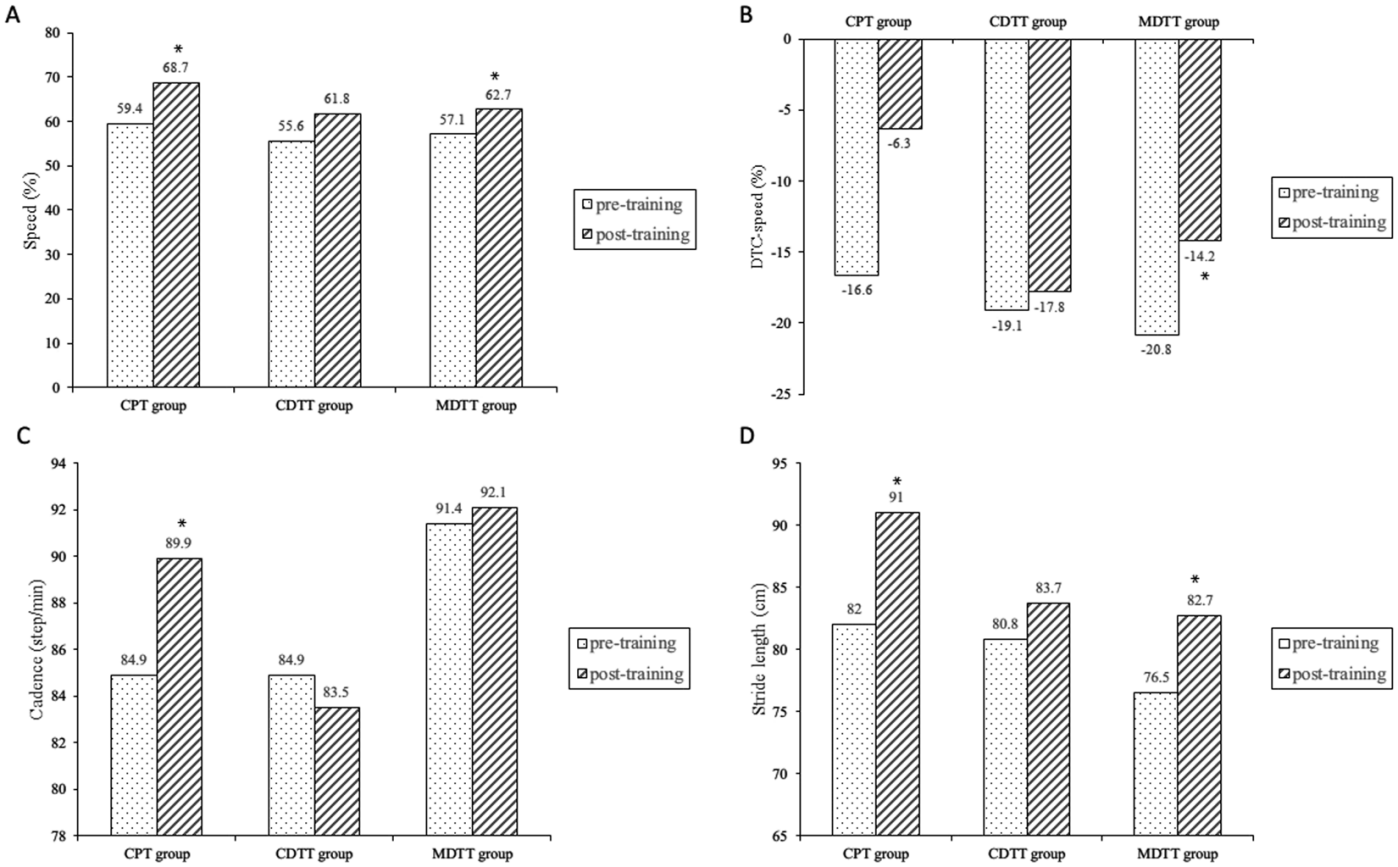
Motor dual task gait performance after different training protocols. (**A**) Speed, (**B**). Dual task cost-speed, (**C**). Cadence, (**D**). Stride length*, significant intra-group difference p < 0.05. Abbreviations: CPT, conventional physical therapy; CDTT, cognitive dual task gait training; MDTT, motor dual task gait training. DTC-speed, dual task cost of speed.

Regarding motor dual task gait performance (walking while carrying a tray), significant improvements in gait speed (p = 0.008), DTC-speed (p = 0.008), and stride length (p = 0.008) were found in the MDTT group compared with pre-training measurements (Fig. 3). In the CPT group, significant increases from pre-training measurements were observed in gait speed (p = 0.028), cadence (p = 0.028), and stride length (p = 0.022) (Table 3 and Fig. 3). However, the insignificant decrease (p = 0.068) in DTC-speed (10.3%) while motor dual task walking after CPT may be due to the high standard deviation (15.8%).

**Table 3:** Motor dual task gait performance after different training protocols.

	CPT group (n = 10)		CDTT group (n = 9)		MDTT group (n = 9)	
	Pre	Post	Pre	Post	Pre	Post
Speed (cm/sec)	59.4 ± 24.5	68.7 ± 24.5*	55.6 ± 14.7	61.8 ± 21.8	57.1 ± 12.0	62.7 ± 12.5*
Change values^a^		9.3 ± 10.5		6.2 ± 10.5		5.6 ± 2.6
Dual task cost-speed (%)	−16.6 ± 13.0	−6.3 ± 8.2	−19.1 ± 10.0	−17.8 ± 12.7	−20.8 ± 9.0	−14.2 ± 9.4*
Change values^a^		10.3 ± 15.8		1.3 ± 7.2		6.5 ± 4.3
Cadence (step/min)	84.9 ± 15.9	89.9 ± 15.9*	84.9 ± 12.1	83.5 ± 21.8	91.4 ± 9.5	92.1 ± 9.1
Change values^a^		5.0 ± 6.8		−1.4 ± 16.0		0.7 ± 1.9
Stride time (sec)	1.46 ± 0.27	1.37 ± 0.25	1.43 ± 0.21	1.40 ± 0.24	1.33 ± 0.13	1.31 ± 0.13
Change values^a^		−0.09 ± 0.14		−0.03 ± 0.15		−0.01 ± 0.03
Stride length (cm)	82.0 ± 20.1	91.0 ± 19.7*	80.8 ± 14.8	83.7 ± 17.8	76.5 ± 17.0	82.7 ± 16.9*
Change values^a^		9.1 ± 11.7		3.0 ± 6.4		6.2 ± 2.8

Abbreviations: CPT, conventional physical therapy; CDTT, cognitive dual task gait training; MDTT, motor dual task gait training.

Values are mean ± SD.

*p < 0.05 for intra-group difference.

^a^Change values were calculated by subtracting the pre-training data from the post-training data.

The results of single walking performance after training are shown in Table 4. After MDTT, the decrease in cadence (p = 0.011) and increases in stride time (p = 0.021) and stride length (p = 0.021) were significant compared to pre-training measurements (Table 4). Moreover, cadence was increased by 4.3 ± 6.1 steps/min in CDTT group, significantly greater than the CPT group (p = 0.011) and MDTT group (p = 0.005).

**Table 4:** Single walking gait performance after different training protocols.

	CPT group (n = 10)		CDTT group (n = 9)		MDTT group (n = 9)	
	Pre	Post	Pre	Post	Pre	Post
Speed (cm/sec)	71.1 ± 20.8	73.7 ± 23.8	70.6 ± 17.7	74.9 ± 20.7	71.8 ± 10.6	72.8 ± 9.8
Change values^a^		2.7 ± 6.1		4.3 ± 6.6		0.9 ± 2.3
Cadence (step/min)	90.9 ± 13.3	89.7 ± 15.2	90.31 ± 10.9	94.6 ± 10.9	96.3 ± 8.4	93.8 ± 6.6*
Change values^a^		−1.1 ± 3.2		4.3 ± 6.1^+^		−2.5 ± 3.1
Stride time (sec)	1.35 ± 0.22	1.27 ± 0.20	1.35 ± 0.17	1.29 ± 0.16	1.25 ± 0.10	1.28 ± 0.10*
Change values^a^		−0.08 ± 0.27		−0.06 ± 0.11		0.03 ± 0.03
Stride length (cm)	93.5 ± 16.29	97.6 ± 19.1	94.1 ± 17.8	95.1 ± 20.1	90.6 ± 14.8	94.4 ± 13.6*
Change values^a^		4.1 ± 7.4		1.0 ± 5.5		3.8 ± 3.8

Abbreviations: CPT, conventional physical therapy; CDTT, cognitive dual task gait training; MDTT, motor dual task gait training.

Values are mean ± SD.

*p < 0.05 for intra-group difference, ^+^p < 0.05 compared with motor dual task group and control group.

^a^Change values were calculated by subtracting the pre-training data from the post-training data.

## Discussion

This randomized controlled pilot trial is the first study to compare the effects of different types of dual task gait training on dual task gait performance in individuals with stroke. To date, only few studies emphasized the effects of cognitive dual task gait training on dual task gait performance. In current study, we found significant improvement in DTC-speed and increase in stride length during cognitive-motor dual task walking after CDTT in stroke patients. Plummer *et al*. also demonstrated improvements in cognitive-motor dual task walking speed after gait training concurrently with cognitive task^22^. However, in current study, we did not find significant improvements in single walking or motor dual task walking performance after CDTT. These results suggest training-specific improvements.

In current study, MDTT was also found to have significant training-specific effects. Significant improvements in gait speed and stride length during motor dual task walking were found after MDTT and consistent with findings in a previous study^21^. Moreover, we further assessed deterioration of gait performance during dual task test conditions relative to a single task by calculating the DTC-speed. The DTC has recently come into widespread use as a measure of dual task interference^23–25^. In current study, we found relative change of 31% in cognitive-motor DTC-speed after CDTT and 31% in motor DTC-speed after MDTT, compared with pre-training measurements. Although there was no significant inter-group difference after intervention, these findings seem to suggest that different types of dual task gait training can be applied to target and improve specific dual task interference, and thereby enhance stroke subjects’ ability to perform different types of dual tasks.

On the other hand, gait speed, cadence, and stride length were all found to improve during motor dual task walking after conventional physical therapy training. To our knowledge, this is the first study to show that conventional physical therapy may result in improved motor dual task gait performance in stroke subjects. Several studies have reported benefits of cognitive-motor dual task walking from exercise interventions in elderly subjects. Cadore *et al*. found significant improvement in cognitive-motor dual task walking performance after participation in a multi-component exercise program including muscle strengthening, balance, and gait training in institutionalized, frail nonagenarians^26^. Hiyama *et al*. found that four weeks of conventional physical therapy plus walking training improved cognitive-motor dual task gait performance (walking while performing serial subtraction) in elderly subjects with knee osteoarthritis^27^. The capacity-sharing theory provides one possible explanation for these findings. In the setting of limited attentional resources, if two attention-demanding tasks are performed at the same time, performance on at least one of the tasks will deteriorate^28, 29^. We speculate that our conventional physical therapy intervention, including muscle strengthening, balance, and gait training, might have improved motor capacity and reduced the attention needed to perform the motor task, permitting greater attention to be directed toward performing other concurrent tasks. The decrease in motor DTC-speed by 10.3% after CPT may also reflect partly the above mentioned speculation. However, this capacity-sharing theory is not supported by our findings for cognitive-motor dual task walking condition.

Moreover, whether the acquisition of dual tasking ability can transfer to untrained tasks has also been studied. Improvements in untrained dual task performance after dual task training have been described in elderly subjects^13, 30, 31^ and in individuals with Parkinson’s disease^32, 33^. In the present study, the test conditions for motor and cognitive dual task gait performance (tray carrying while walking and serial subtraction while walking respectively) were novel to all participants. For individuals with stroke in current study, our results demonstrate positive transfer effects for untrained motor and cognitive-motor dual tasking after dual task gait training interventions. These results contrast with the findings of Plummer *et al*., who reported low transfer effects for untrained tasks in subjects with stroke^22^. One possible reason for this discrepancy in findings may be differences in the dual task challenges that were studied as outcomes. The training tasks in our study and Plummer’s study were both cognitive executive function tasks. However, while the current study used an executive function task as an outcome measure (cognitive-motor dual task gait performance), in Plummer’s study, a visuospatial task was used to assess transfer effects of the training intervention. It is possible that positive transfer effects may be observed for tasks of the same type but not occur as efficiently for different types of cognitive tasks.

In addition, in current study we found that CDTT was associated with a greater increase in cadence during single walking when compared to CPT or MDTT. However, gait speed, the most important objective clinical measure of functional ability, was not significantly different. These findings suggest that the different types of gait training included in current study (cognitive dual task gait training, motor dual task gait training, and conventional physical therapy) may all positively impact single walking performance but through different effects on walking characteristics.

The results of this study suggest that cognitive and motor dual task gait training may be incorporated into stroke rehabilitation protocols to improve dual task gait performance. However, there are some limitations to this study. First, the sample size is relatively small. A larger, randomized controlled clinical trial is needed to validate the reported benefits of the dual task gait training protocols reported in current study. Second, in current study, the therapist was not blinded to the group assignments. Although unavoidable, this lack of blinding might be a source of bias for the better results of dual task gait training. Third, the short intervention period of 4 weeks in current study should also be noted. In addition, we did not measure the single cognitive or motor task performance (e.g. accuracy or success) during the dual task walking assessment. Therefore, interactions between gait performance and single cognitive or motor task performance were not evaluated. Finally, this study did not provide the follow-up effects, therefore we could not demonstrate whether these changes could be maintained.

In summary, it seems that CDTT can improve the cognitive dual task gait performance and MDTT can improve the motor dual task gait performance based on several significant intra-group differences. Moreover, these dual task gait training protocols may be easily implemented as part of stroke rehabilitation therapy. Therefore, different types of dual task gait training can be adopted to enhance different dual task gait performance in individuals with stroke. For subjects recovering after stroke, dual task gait training offers particular promise for supporting return to more complex community activities.

## Methods

### Participants

The study protocol was approved by the Institutional Review Board of Taipei Veterans General Hospital and of National Yang-Ming University. The approved protocol was followed throughout the study period. This trial was registered at http://www.anzctr.org.au/ (ACTRN12616000225415 on February 18, 2016) and conformed to the CONSORT checklist. Participants were recruited from a medical center and the community between October 2013 and October 2015 in Taiwan. Inclusion criteria for this study were the following: (1) hemiparesis from a single stroke; (2) age between 20 to 80; (3) gait velocity sufficient for at least limited community walking ability (35 m/min) by Perry *et al*.’s classification system^34^; (4) able to walk 10 m independently without an assistive device; (5) able to use the non-affected upper extremity to hold a tray to complete the assessment; (6) stable medical condition allowing participation in the testing protocol and intervention; and (7) a score of greater than 24 on the mini-mental state examination (MMSE). Exclusion criteria were (1) any comorbidity or disability other than stroke that would preclude gait training; (2) any uncontrolled health condition for which exercise was contraindicated; and/or (3) any neurological or orthopedic disease that might interfere with the study. In total, 44 individuals were identified as potential subjects. Of these, 28 participants provided informed consent for participation in this study (Fig. 1).

### Study Protocol

This study was a single-blinded, randomized controlled trial. An individual who was not involved with the study selected sealed envelopes to assign participants to one of the three treatment groups: cognitive dual task gait training (CDTT), motor dual task gait training (MDTT), or conventional physical therapy (CPT). All training sessions were 30 minutes long, and every training was administered three sessions per week for a total of four weeks by the same physical therapist. All outcomes were measured on the day before the training intervention began (pre) and on the day after the intervention was completed (post) (Fig. 1).

### Measurements

For each participant, gait performance was measured in three conditions: (1) single comfortable walking (single motor task), (2) comfortable walking while serial subtracting by three, starting from a randomized 3-digit number (e.g. 100, 97, 94…) (cognitive-motor dual task), and (3) comfortable walking while carrying a tray with a bottle of water in front of the subject with the non-affected hand (motor dual task). Testing was performed in random order to minimize the effects of practice or fatigue, and each condition was measured twice (one minute per trial) with one minute rest in between. Therefore, the total time covered of the testing conditions was about 13 min. The average value for each measured outcome variable was used for data analysis.

Gait parameters measured for the above test conditions were obtained using a GAITRite system (CIR system, Inc., Havertown, Pennsylvania). The GAITRite system is a straight walkway containing pressure-sensitive sensors. The walkway is 4.75 m long and 0.9 m wide, and the pressure-sensitive area is 4.30 m long and 0.61 m wide. When a subject ambulates along the walkway, the contact time and location of each footfall are recorded and analyzed on a laptop using Microsoft Excel 2013 to calculate temporal-spatial parameters of walking. The concurrent validity and reliability of the GAITRite system have been previously established in healthy young, elderly adults^35, 36^, and stroke subjects^37^. Also, the test-retest reliability of the GAITRite system in stroke subjects while executing dual tasks has been proven^38^. Gait parameters recorded during each trial were speed, cadence, stride time, and stride length. Dual task interference is quantified by calculating the dual task cost of gait speed (DTC-speed)^23–25^, was calculated using the following formula: (dual task walking speed – single task walking speed)/single task walking speed * 100%^39^.

### Intervention

Participants in the CDTT group were instructed to perform cognitive tasks during diverse walking conditions on a level surface. The cognitive dual tasks included: (1) walking while repeating phrases; (2) walking while counting numbers forward in order by ones; (3) walking while counting numbers backward in order by ones; (4) walking while executing a word chain (i.e. speaking out word that begins with last letter of previous word); (5) walking while reciting a poem; (6) walking while talking; and (7) walking while reciting a sentence backward. Practices for these comfortable walking conditions included walking forward, walking backward, and walking on an S-shaped route. Participants were challenged with increasingly difficult tasks as the training progressed.

Participants in the MDTT group were instructed to perform motor tasks during diverse walking conditions on a level surface. The motor dual tasks included: (1) walking while holding one or two balls (diameter = 20 cm)^21^; (2) walking while raising an umbrella using both hands; (3) walking while waving a rattle; (4) walking while beating a castanet; (5) walking while bouncing a basketball (diameter = 24.6 cm)^21^; (6) walking while kicking a basketball which was in a net held by the participant^21^; and (7) walking while holding one ball and concurrently kicking another basketball in a net^21^. Practices for these comfortable walking conditions included walking forward, walking backward, and walking on an S-shaped route. Participants were challenged with increasingly difficult tasks as the training progressed.

During training session, subjects in CDTT group and MDTT group were asked to focus on “both” tasks as possible during performing the dual task gait exercises. In addition, the difficulty of exercise was gradually increased. For example, the exercise started first with cognitive (motor) task 1 while walking forward, progressed to walking backward and walking on the S-shaped route, and then with cognitive (motor) task 2 while walking forward, progressed to walking backward and walking on an S-shaped route, and so on.

Participants in the CPT group received conventional physical therapy, which included single focus training activities for muscle strengthening, balance, and gait. The key muscles strengthened included hip flexors, hip extensors, hip abductors, knee extensors, knee flexors, ankle dorsiflexors and ankle plantarflexors. The balance training included (1) weight shifting exercise to different directions while standing, (2) squatting against a gymnastic ball, (3) standing on a foam with eyes open/close, (4) tandem standing with eyes open/close, and (5) single leg standing. The gait training included walking forward, walking backward, and walking on an S-shaped route.

### Data Analysis

Descriptive statistics (mean ± standard deviation or frequency) were generated for all variables. Intergroup differences among baseline characteristics were evaluated using Kruskal-Wallis one-way analysis of variance by ranks or χ^2^ analysis. Intragroup difference was analyzed by Wilconxon sign ranks test to determine the improvement after training. Change values for the outcome measures were calculated by subtracting the baseline data from the post-intervention data. To analyze intergroup improvement, the change values were analyzed using Kruskal-Wallis one-way analysis of variance by ranks with group as a factor. Statistical significance was set as p < 0.05.
